# Electrospinning Fabrication and Cytocompatibility Investigation of Nanodiamond Particles-Gelatin Fibrous Tubular Scaffolds for Nerve Regeneration

**DOI:** 10.3390/polym13030407

**Published:** 2021-01-27

**Authors:** Elena Olăreț, Diana-Maria Drăgușin, Andrada Serafim, Adriana Lungu, Aida Șelaru, Alexandra Dobranici, Sorina Dinescu, Marieta Costache, Iulian Boerașu, Bogdan Ștefan Vasile, Doris Steinmüller-Nethl, Horia Iovu, Izabela-Cristina Stancu

**Affiliations:** 1 Advanced Polymer Materials Group, University Politehnica of Bucharest, 011061 Bucharest, Romania; elena.olaret@upb.ro (E.O.); diana.m.dragusin@gmail.com (D.-M.D.); andrada.serafim@gmail.com (A.S.); adriana_lungu2006@yahoo.com (A.L.); horia.iovu@upb.ro (H.I.); 2 Department of Biochemistry and Molecular Biology, Faculty of Biology, University of Bucharest, 050095 Bucharest, Romania; aida.selaru@bio.unibuc.ro (A.Ș.); dobranici.alexandra-elena@s.bio.unibuc.ro (A.D.); sorina.dinescu@bio.unibuc.ro (S.D.); marietacostache@gmail.com (M.C.); 3 Department of Immunology, National Institute for Research and Development in Biomedical Pathology and Biomedical Sciences “Victor Babes”, 050096 Bucharest, Romania; 4 The Research Institute of the University of Bucharest, 050663 Bucharest, Romania; 5 National Research Center for Micro and Nanomaterials, Faculty of Applied Chemistry and Materials Science, University Politehnica of Bucharest, 060042 Bucharest, Romania; iulianboerasu@gmail.com (I.B.); vasile_bogdan_stefan@yahoo.com (B.Ș.V.); 6 National Research Center for Food Safety, Faculty of Applied Chemistry and Materials Science, University Politehnica of Bucharest, 060042 Bucharest, Romania; 7 DiaCoating. GmbH, 6112 Wattens, Austria; doris.steinmueller@diacoating.com

**Keywords:** electrospun fibers, fish gelatin, diamond nanoparticles, nerve guidance channel

## Abstract

This paper reports the electrospinning fabrication of flexible nanostructured tubular scaffolds, based on fish gelatin (FG) and nanodiamond nanoparticles (NDs), and their cytocompatibility with murine neural stem cells. The effects of both nanofiller and protein concentration on the scaffold morphology, aqueous affinity, size modification at rehydration, and degradation are assessed. Our findings indicate that nanostructuring with low amounts of NDs may modify the fiber properties, including a certain regional parallel orientation of fiber segments. NE-4C cells form dense clusters that strongly adhere to the surface of FG50-based scaffolds, while also increasing FG concentration and adding NDs favor cellular infiltration into the flexible fibrous FG70_NDs nanocomposite. This research illustrates the potential of nanostructured NDs-FG fibers as scaffolds for nerve repair and regeneration. We also emphasize the importance of further understanding the effect of the nanofiller-protein interphase on the microstructure and properties of electrospun fibers and on cell-interactivity.

## 1. Introduction

The self-regeneration ability in nerve injury depends on the severity of the wound and the gap between the two ends. When the defect is small enough (<5 mm) with no tension occurring between the ends, the first approach is the coaptation of the two ends by suture or using fibrin glue [1]. When tension-free is not possible, autografts remain the gold standard in nerve regeneration. Despite obvious advantages, autografts came with some limitations, such as additional surgical intervention, low availability, morbidity, and loss of function of donor-site or mismatches in size. An alternative is represented by the largely available nerve allografts that do not lead to the morbidity of the donor site, although they require administration of immunosuppressive treatment and present high costs. To overcome such drawbacks, decellularized allografts have been produced, while their main advantage is the preservation of the internal structure of the nerve. However, it has been reported that such scaffolds only support a limited range of dimensions: Small diameters (1–2 mm) and 30 cm length [2].

The limited capacity of peripheral nerve injury (PNS) for self-regeneration with a functional outcome in case of the severe lesion and the limitations of the current grafts lead to the development of new strategies for nerve regeneration improvement. Artificial nerve guidance channels (NGCs) are usually biocompatible tubular structures capable to bridge the two ends of the nerve and assuring mechanical stability during the regeneration process. Ideally, NGCs should provide an equilibrium between degradation and healing rates, allow the exchange of nutrients and oxygen, while preventing the infiltration of scar tissue, and may provide guidance cues for axonal growth [3]. Micro- or nanostructured morphology, intraluminal presence of longitudinally oriented elements, or the controlled release of growth factors represent advanced strategies that may improve NGCs performances [4,5,6]. Various polymers have been investigated as scaffolds for neuronal regeneration [7], including natural polymers, such as collagen [8], gelatin [9], fibroin [10], chitosan [11], synthetic polymers, e.g., polycaprolactone [12,13], or hybrid blends, e.g., poly(ethylene glycol)- poly(3-caprolactone) nanoparticles, dispersed in a gelatin-methacryloyl hydrogel [14], and silk sericin/silicon [15]. The strategies to fabricate NGCs range from conventional dip coating [9], and injection molding [8], up to 3D printing [5,12,14] or combined techniques, such as rolled electrospun mesh [10], and electrospun tubular scaffold filled with cryogel [16].

Fish gelatin is a collagen-derived protein well known and intensively explored as a biomaterial, mainly due to its extracellular matrix (ECM)-origin, low gelling temperature, biodegradability and biocompatibility[17,18,19]. Since cells are naturally accustomed to nanotopographical microenvironments, nanostructuring of scaffolds through surface decoration or using embedded nanoparticles was investigated to additionally enhance cellular guidance [20,21]. It has been proved that nanocarbon species, such as graphene, graphene oxide, and nanodiamond particles (NDs), present potential in stimulating interactions with neuronal stem cells (NSCs) [20,21,22]. Previous work of our group reported the in vitro potential of electrospun scaffolds based on FG loaded with NDs to stimulate the cell-interactivity of the matrix [23,24]. While nanocrystalline diamond (NCD) offers the possibility to generate cell growth substrates with well-defined surface properties, the potential of NDs to serve as nanoplatforms with enhanced responsiveness remains underexplored. The present work aimed at the fabrication of fibrous cylindrical scaffolds for potential use in nerve regeneration and explored the responsiveness of neuronal stem cells (NSCs) to the nanofibers of FG seeded with low amounts of NDs.

## 2. Materials and Methods

Cold water fish skin gelatin (FG), phosphate-buffered saline (PBS), absolute ethanol (EtOH), and aqueous glutaraldehyde solution 50% *w/v* (GA) were purchased from Sigma Aldrich. Carboxyl-functionalized nanodiamond nanoparticles (NDs) were kindly provided by DiaCoating (Wattens, Austria). All raw materials were used as received without further purification.

### 2.1. Preparation of Electrospinning Solutions

Two series of electrospinning compositions were prepared to vary the protein concentration, in the presence or absence of NDs. For NDs-containing samples, aqueous nanoparticle suspensions (1% *w/v*) were treated with ultrasound for 30 min. Then, the corresponding amount of protein was added to final concentrations of 50% *w/v* and 70% *w/v*, protein content per volume of NDs suspension. FG dissolution occurred under continuous stirring, at 40 °C. The samples without NDs were prepared to serve as a control. All compositions were kept at 4 °C prior to use.

### 2.2. Fabrication of Fibrous Scaffolds

Composite fibers were obtained through electrospinning by adapting the fabrication protocol described in [25]. Briefly, each solution was loaded in a 12.45 mm diameter syringe supplied with a 0.8 internal diameter needle. The syringe was mounted into a specific pump, which was set to a flow rate of 7 µL/min for all compositions. An electrospinning equipment (EC-CLI, IME Medical Electrospinning, Waalre, The Netherlands) with a cabinet allowing to set the environmental conditions to 25 °C and 40% relative humidity was used. The positive voltage applied was set in between 19 kV and 20 kV depending on the composition in use. A 2 mm diameter cylindrical collector was used, rotating at 70 rpm and placed at 17 cm distance from the needle to obtain the fibrous scaffolds. When the equivalent of 700 µL solution is extruded, the fibers-coated collector is removed and subjected to a crosslinking treatment in the vapor-saturated atmosphere of GA, for seven days, at room temperature. Then, an extensive rinsing in double distilled water is performed for four days, to remove any unreacted components. During this step, the fibrous scaffold also rehydrates while crosslinked, then its ends are removed, allowing for a flawless detachment of the remaining tubular scaffold, which will be further sized and investigated. For cytocompatibility and contact angle studies, the fibrous meshes were fabricated using a 2 cm diameter collector.

### 2.3. Scaffolds’ Characterization

#### 2.3.1. Rehydration Capacity and Dimensional Modification

The rehydration capacity was evaluated by means of maximum swelling degree (MSD). One centimeter length tubular samples were weighed and incubated in PBS pH 7.4 at 37 °C. Every 10 min in the first hour, samples were removed from the incubation medium, gently wiped with filter paper, and weighed. MSD was calculated according to Equation (1), where *m_f_* is the weight for the equilibrium swollen scaffold and *m_i_* is the initial weight of the dry scaffold.

(1) $$\mathrm{MSD},\%=\frac{m_{f}-m_{i}}{m_{i}}\times 100$$

Dimensional modification at rehydration was evaluated, comparing dry samples and swollen samples after maximum swelling degree was reached. In this respect, samples were measured using Image J software.

#### 2.3.2. Degradation Study

Degradation was investigated in static condition by immersing 1 cm length tubular samples in PBS pH 7.4 at 37 °C, for 14 days. Samples were weighed in the dry state (*m_i_*) before and after incubation (*m_f_*), and mass loss (ML) was determined according to Equation (2). The released protein fraction (RPF) from incubation media was spectrophotometrically quantified using the micro-bicinchoninic acid (BCA) assay kit, which has a sensitivity down to 0.5 µg/mL. In brief, 150 µL of working reagent (WR) (prepared accordingly to BCA assay working protocol) and 150 µL immersion media were mixed and incubated for two hours at 37 °C. A dilution scheme for the immersion media and protein samples of known concentration was also prepared. A Tecan Infinite M200 PRO spectrophotometer (Tecan, Männedorf, Switzerland) with the 96-well plate reader was used to quantify the absorbance at 562 nm. The absorbance was measured in four different places of each well, and the averaged value was further used. The protein concentration of the unknown sample (*C*) was determined from the standard curve, and the amount of solubilized protein (*m_p_*) was computed according to Equation (3), where *V_s_* is the volume of the tested sample and *df* is the dilution factor. Then, RPF was estimated according to Equation (4), where *m_p_* is the total amount of solubilized protein from the degradation medium and *m_i_* is the corresponding initial weight of the sample (NDs weight was considered negligible).

(2) $$\mathrm{ML},\%=\frac{m_{i}-m_{f}}{m_{i}}\times 100$$

(3) $$m_{p}\;\left(mg\right)=\;C\times V_{s}\times df$$

(4) $$RPF,\;\%=\frac{m_{p}}{m_{i}}\times 100$$

The relevance of static conditions is often considered limited with respect to real in vivo conditions. Therefore, ML was estimated through accelerated degradation performed in a 3DCulturePro™ Bioreactor (TA Instruments, Eden Prairie, MN, USA), which provides flow around and through the sample. For an accelerated degradation, the experiment was conducted for 14 days, in PBS 7.4, at 37 °C, with a flow rate of 0.76 mL/min. ML was gravimetrically determined according to Equation (2), where *m_i_* is the mass of the dried sample before incubation and *m_f_* is the mass of the dried sample after degradation.

#### 2.3.3. Contact Angle Measurements

Wettability of the 2D fibrous substrate was monitored using a Drop Shape Analyzer 100 (DSA 100, KRÜSS GmbH, Hamburg, Germany). Two microliters of distilled water were dropped on the mesh surface, and contact angle was recorded for 260 seconds. All experiments were performed in triplicate.

#### 2.3.4. Architectural and Microstructural Characterization

The fibrous scaffolds were investigated through environmental scanning electron microscopy (ESEM), and atomic force microscopy (AFM). A focused ion beam-scanning electron microscope (FIB-SEM) system, model Versa 3D (Thermo Fisher Scientific, Co., Waltham, MA, USA), was involved in investigating the cell–cell and cell–mesh interaction on the prepared set of samples. In order to minimize the sample damage through the electron beam interaction, the samples’ surface (0° tilt) was investigated in High-Vacuum (6.1E-4 Pa) at a working distance of 9 mm, using 10kV as accelerating voltage. Prior to SEM investigation, a thin gold (Au) conductive coating was sputtered all-over the sample, to avoid the static charge build-up. The topographical and compositional information were concluded by simultaneously recording the secondary electrons (SE) and backscattered electrons (BSE) signals through the Versa 3D specialized ETD and CBS detectors.

NDs distribution within the fibers was investigated through transmission electron microscopy (TEM) using a TECNAI F30 G2 S-TWIN microscope (Fei, Hillsboro, Oregon, USA) operated at 300 kV with EDX and EELS facilities, as described in [24]. Briefly, a small piece of scaffolds was placed on a TEM copper grid and covered with amorphous C film with holes. Additionally, FG_NDs fibers surface were observed in air, at room temperature using a tapping mode AFM module from a neaSNOM equipment (Neaspec, Martinsried, Germany) also. A standard Pt/Ir coated (Arrow™ NCPt, NanoWorld, Neuchâtel, Switzerland) AFM tip with a radius less than 25 nm was used for image acquisition.

#### 2.3.5. Cytocompatibility Evaluation

To explore the biological effect of NDs on the cytocompatibility of the electrospun meshes, they were tested with murine neural stem cells (mNSCs). The samples were sterilized by exposure to UV light. The materials were then placed in 24-well culture plates and exposed to a complete culture medium (supplemented with 10% fetal bovine serum (FBS) and 1% antibiotic) overnight.

##### Achievement of 2D Cell-Scaffold System

Neural progenitor cells from NE-4C cell line (ATCC, CRL-2925) were grown and expanded in culture following the manufacturer’s instructions, in Eagle’s Minimum Essential Medium (EMEM), supplemented with L-glutamine and 10% fetal bovine serum (FBS). Cells in passage 2 were seeded on FG-based meshes in 24-well culture plates at a density of 2.5 × 10^4^ cells/cm^2^, thus resulting in cell-scaffold systems (further named NE-4C/FG_NDs and NE-4C/FG). These were maintained in standard culture conditions (37 °C, 5% CO_2_, and humidity) for seven days, during which cytocompatibility assays were carried out at 2- and 7-days post-seeding.

##### The Evaluation of In Vitro Cytocompatibility in NE-4C/FG Systems

##### MTT Assay

The metabolic activity of living cells in contact with FG-based meshes was assessed using methylthiazolyldiphenyl tetrazolium bromide (MTT, Sigma-Aldrich Co, Steinheim, Germany). The solution was prepared at the recommended concentration of 1 mg/mL in culture media lacking FBS. After 4 h of incubation with MTT solution, the resulted formazan crystals were dissolved using isopropanol to a final violet solution. Neural progenitors seeded at a density of 2.5 × 10^4^ cells/cm^2^ on 24-well plates served as a positive control of cell viability. The obtained solution was measured at 550 nm using FlexStation3, Molecular Devices, USA spectrophotometer.

##### LDH Assay

The cytotoxic effect of the scaffold was assessed using the “In vitro toxicology assay kit lactate dehydrogenase based” TOX7 kit (Sigma Aldrich Co, Steinheim, Germany). The positive control for this assay was represented by NE-4C cells seeded on the culture dish, which were treated with 2% Triton-X100 (Sigma/Merck, Steinheim, Germany) in order to induce 100% of cell cytotoxicity. The test was performed following the manufacturer’s instructions, and the final product was measured at 490 nm, using FlexStation3 spectrophotometer, Molecular Devices, San Jose, CA, USA.

##### Live/Dead Staining

To qualitatively observe the living and dead cells in contact with the scaffolds, the staining was performed using a Live/Dead kit (Invitrogen, Life Technologies, Foster City, CA, USA). The staining solution was prepared following the manufacturer’s instructions, and 300 µL of staining solution was added to each well and incubated for 20 min in the dark, at room temperature. The examination was performed using a laser-scanning confocal microscope (Nikon A1/A1R Confocal Laser Microscope System, Tokyo, Japan), and images were analyzed using the corresponding software.

### 2.4. Statistical Analyses

All experiments were performed in triplicate (n = 3), and the results were expressed as a ± standard deviation (SD) using GraphPad Prism Software 6.0 (GraphPad Software Inc., San Diego, CA, USA). Statistical relevance was assessed using the same software, one-way ANOVA method, and Bonferroni post-test, and significant statistical differences were considered for *p* < 0.05.

## 3. Results

### 3.1. Fabrication and Characterization of Fibrous Tubular Scaffolds

The potential of NDs to guide or stimulate cell adhesion as films or when dispersed at low loadings (below 3%) in fibrous scaffolds based on FG was previously reported [23,24,26]. To investigate the effect of low amounts of NDs on cell-interactions, the protein was dissolved in 1% nanoparticle dispersions. Two types of fibrous scaffolds were fabricated through electrospinning: FG50_NDs with a final protein concentration of 50% and FG70_NDs with 70% FG. The control FG50 and FG70 scaffolds without NDs were also fabricated.

**Figure 1:**
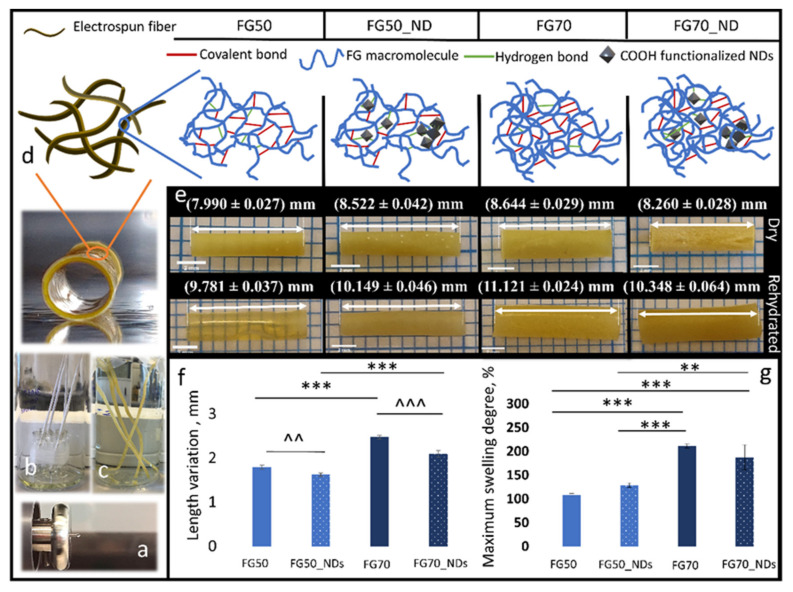
Electrospinning fabrication of tubular fibrous scaffolds: (**a**) Solution injection; (**b**) collectors coated with deposited fibers, prepared for crosslinking; (**c**) crosslinked meshes after the crosslinking treatment; (**d**) transversal view of a crosslinked tubular scaffold after its removal from the collector and schematic representation of the crosslinked polymer macromolecules with embedded NDs or without them; (**e**) representative digital photos of dry (upper raw) and rehydrated (bottom raw) tubular samples (from left to right FG50, FG50_NDs, FG70, FG70_NDs), all values are expressed in mm; (**f**) length variation at rehydration, and (**g**) maximum swelling degree, statistical significance: ^^,** *p* = 0.001, ^^^,*** *p* < 0.001.

Figure 1a depicts the steps of the fabrication of tubular scaffolds with diameters around 2 mm using electrospinning. The injected solution (Figure 1a) forms white meshes homogeneously covering the collector (Figure 1b). Their appearance changed from white to yellowish following the crosslinking treatment (Figure 1c). Hydrogen bonds and chemical crosslinks interconnect FG macromolecules or FG and NDs nanoparticles as schematically displayed in Figure 1d.

Figure 1e presents the four types of tubular scaffolds in a dry or rehydrated state. Swelling is accompanied by low mass and dimensional changes, and the hydrated scaffolds are elastic (Figure 1e–g). It may be noticed that NDs addition reduces the length variation at rehydration (*p* = 0.001 when FG50 and FG50_NDs are compared and *p* < 0.001 when FG70 and FG70_NDs are compared). Such a reinforcing effect was expected, since carboxylated NDs play the role of nanoplatforms for dense hydrogen bonding between their -COOH groups and FG side chains (terminated in -OH and -NH_2_ groups). Moreover, the crystalline NDs, even if used in low concentration, enhances the density of the corresponding fibers when compared to protein networks alone. Considering the aqueous media affinity, no significant influence was found between FGs and FG_NDs samples, while between FG50s and FG70s, differences were significant (*p* < 0.001). Hence, the addition of the NDs nanofiller does not significantly influence the swelling capacity, while the protein content does. The higher the protein amount, the higher the swelling capacity.

**Figure 2:**
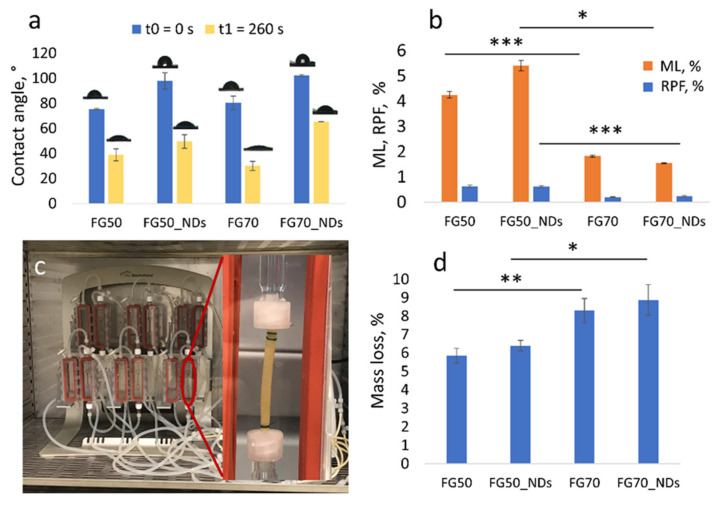
(**a**) Graphical representation of contact angle values immediately after drop deposition and 260 seconds later; (**b**) ML over 14 days of incubation in PBS at 37 °C in static conditions (orange) and RPF through BCA assay (blue); (**c**) representative image of the experimental setup using bioreactor-dynamic conditions, (**d**) graphical representation of samples’ ML over 14 days of testing in dynamic conditions; statistical significance: * *p* < 0.05, ** *p* < 0.01, *** *p* < 0.001.

As depicted in Figure 2b, samples with lower content of protein, FG50 and FG50_NDs presented higher degradation when compared to FG70 and FG70_NDs, during 14 days of evaluation under static testing. This behavior was confirmed by both mass loss and released protein from the scaffolds. It was noticed that FG content was found to significantly influence the degradation under static conditions: The higher the protein content, the lower the degradability of the crosslinked scaffolds, as can be seen in the ML values in Figure 2b. It was observed that the addition of NDs has no significant influence on samples’ stability over 14 days. ML values for each composition are higher than the corresponding RPF, due to the fact that the gravimetric approach quantified macroscopically visible fragments, while BCA method quantified only solubilized protein. The relevance of static conditions is often considered limited with respect to real in vivo conditions. For example, a study on the development of bioreactors for nerve regeneration investigated the effect of flow rates of 0.8 mL/h (0.013 mL/min), 3.0 mL/h (0.05 mL/min), and 5 mL/h (0.08 mL/min) on cell viability and relative cell number [27]. Therefore, additional information on the degradation behavior was obtained through 14 days of incubation using a perfusion bioreactor system. A flow rate of 0.76 ml/min was chosen to provide an accelerated mass loss. ML values increased when compared to the static testing, to 5.86% ± 0.39% for FG50, 6.4% ± 0.28% for FG50_NDs, 8.3% ± 0.66% for FG70 and 8.88% ± 0.84%for FG70_NDs. Interestingly, for the accelerated degradation test, the mass loss is stronger the higher the protein content in the samples (Figure 2d).

NDs addition leads to a lower wettability of both FG systems, as indicated by the contact angle determination (Figure 2a). As expected, the samples are hydrophilic. Since the two series contain only low amounts of nanofiller, water affinity does not significantly vary, as reflected by swelling in Figure 1g, with a slight decrease recorded for FG70_NDs.

### 3.2. Microstructural Characterization

**Figure 3:**
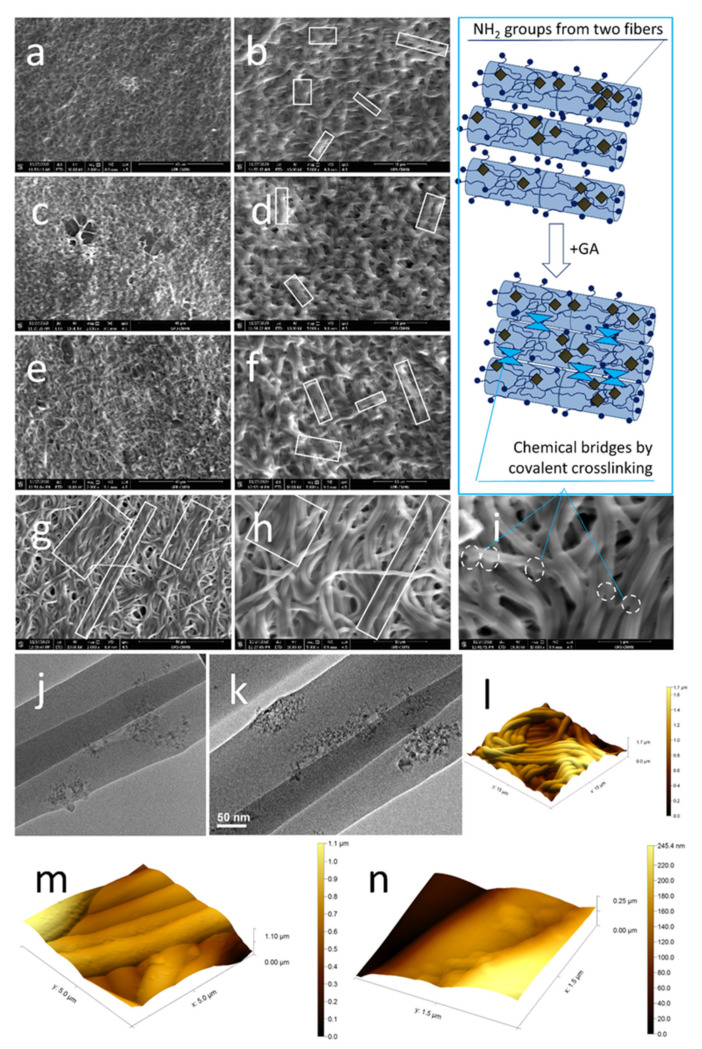
Representative microstructural details of the fibrous: SEM micrographs of (**a**,**b**) FG50, (**c**,**d**) FG50_NDs, (**e**,**f**) FG70, (**g**,**h**) FG70_NDs (white rectangles present parallel-oriented fiber bundles; scale bars 40 µm in (**a**,**c**,**e**,**g**) and 10 µm in (**b**,**d**,**f**,**h**); (**i**) FG70 microstructural detail revealing fibers fusion areas (circles) scale bar 5 µm and schematic representation of covalent crosslinking of amine groups from two fibers; TEM micrographs: (**j**) FG70_NDs, (**k**) FG50_NDs, (**l**–**n**) AFM micrographs of FG70_NDs, with visible NDs-nanostructured surface areas alternating with smooth FG continuous phase.

The SEM micrographs in Figure 3 present dense fibrous meshes with some composition-specific features. Individual fibers seem homogeneous and defect-free. Small pores are formed between entangled fibers. The meshes present entangled fiber bundles formed by 3–5 sub-micron fibers with local parallel orientation not longer than 10 microns, as visible in white rectangles from Figure 2b,d,f,h. Adding NDs lead to no significant morphological difference for FG50 scaffolds. Interestingly, FG70_NDs fibers present stronger longitudinal alignment up to about 60 microns long, as seen in Figure 3g. Increasing protein concentration from 50% to 70% leads to higher total solid content in the fibers. This is reflected in higher dimensions of the fibers richer in protein: FG70 with average diameters around 500 nm and FG50 of about 400 nm. While no visible change was noticed when NDs are added in FG50, with FG50_NDs diameter values around 400 nm, the diameters of FG70_NDs nanocomposite fibers were significantly higher, reaching about 680 nm.

TEM micrographs identified two phases along the fibers, with no visible individual nanoparticles, but rather with NDs agglomerations forming crystalline nanostructured areas into the amorphous continuous polymer phase (Figure 3j,k). Since the nanoparticles were used at low concentration, it was expected that they are not continuously dispersed in the fibers, but rather as small agglomerates placed at different distances. The distribution of the nanostructures within the polymer depends on different factors, including hydrogen bonds formed between the -COOH groups at the surface of NDs and -OH and -NH_2_ groups from FG macromolecules. Moreover, since the electrospinning solution of the FG70 fibers contains the same number of nanoparticles and a higher amount of protein, it was expected a different distribution of the NDs areas, separated by a denser FG network.

The entangled fiber bundle microstructure and the regional NDs nanostructuring were also revealed through AFM (Figure 3l–n). The roughness of the sample is an interesting geometrical/topographical feature that might impact cellular behavior, since it results from fibers entanglement. It should be mentioned here that electrospun fibers, even when crosslinked, remain flexible, since they are water-insoluble while containing a high amount of water, and they are locally bridged through physical fusion and potential local crosslinking of macromolecules at the fiber junctions’ areas (Figure 3i). This suggests a potential time-dependent modification of the roughness and mesh porosity in hydrated media in the presence of cells.

### 3.3. Evaluation of In Vitro Cytocompatibility in NE-4C/FG Systems

**Figure 4:**
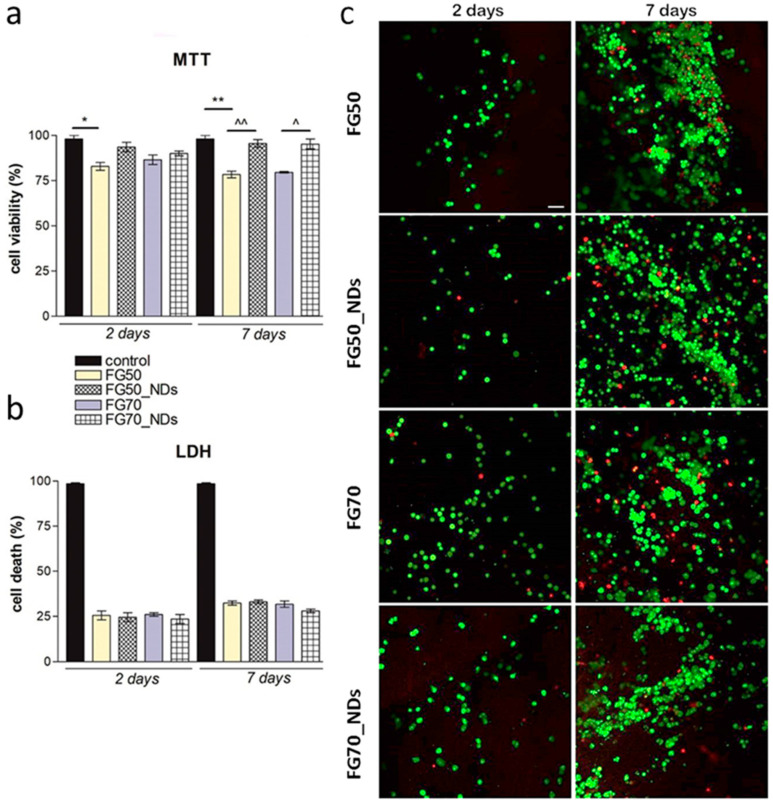
Cytocompatibility evaluation of FG scaffolds in contact with neural stem cells. (**a**) Evaluation of cells metabolic activity by MTT assay during one week of in vitro culture; (**b**) evaluation of the materials’ cytotoxicity as revealed by LDH test after 2 and seven days of cell culture; statistical significance * and ^*p* < 0.05, ** and ^^*p* < 0.01; (**c**) Live/Dead staining on NE-4C/FG systems, shown through using a confocal microscopy: live (green labeled with calcein AM) and dead (red labeled with ethidium bromide) cells. Scale bar = 50 µm.

MTT profile (Figure 4a) revealed that at two days post-seeding, cells on all composites maintained a good viability rate in the range of 83–93% and no significant changes between the four different tested materials were registered. Even so, slightly higher viability could be observed on the meshes loaded with 1% NDs (93% and 90% respectively, as compared to 83% and 85%), while the lowest viability rate was registered on FG50 materials (83%). At seven days of culture, cells’ metabolic activity was found to be significantly higher (*p* < 0.01) on FG50 composite S loaded with 1% NDs as compared to the corresponding control (FG50) (95% compared to 78%). No significant differences were registered between NE-4C/FG50 and NE-4C/FG70, suggesting that the addition of more gelatin to the composition, has no significant influence upon cell viability.

During the assessment of cell viability, a reference was considered, represented by a bidimensional culture of NE-4C seeded on 24-well plates where the viability was 98%. In contrast, the additional reference used for the cytotoxicity assay was represented by a culture of neural progenitors exposed to Triton-X100 in order to report the data to a 100% dead (cytotoxic) control. This allowed cytotoxicity data representation in % related to this positive control (Figure 4b). LDH (Figure 4b) indicated reduced amounts of toxicity after two days of culture on all tested composites. These remained quite constant during one week of culture, with no significant changes found between the materials. It can be noticed that compared to the positive control, all tested composites presented a significantly low percentage of cytotoxicity during one week of cell culture. These results indicate that fish gelatin composites loaded with 1% NDs do not induce significant cell death on murine neural stem cells during one week of culture.

Images obtained after examination in confocal microscopy (Figure 4c) revealed a high proportion of live cells in contact with FG meshes at two days post-seeding, strengthening the hypothesis that these materials are able to support cell viability. Interestingly, from two to seven days of culture, cells proliferated on all tested composites, especially on those enriched with 1% NDs, indicating that the presence of NDs in the materials’ composition supports cell viability and enhances cell proliferation. It is important to mention that cells formed large groups on all tested composites, indicating that these scaffolds offer optimal adhesion and growth conditions for murine neural stem cells. Slightly higher proliferation was found on FG70 materials, suggesting that the higher the amount of gelatin offers more favorable conditions, but not in a significant manner, as compared to FG50 materials. Thus, this assay confirms the results obtained by MTT and LDH assay, making these NDs-loaded meshes a potential candidate for peripheral nerve regeneration.

**Figure 5:**
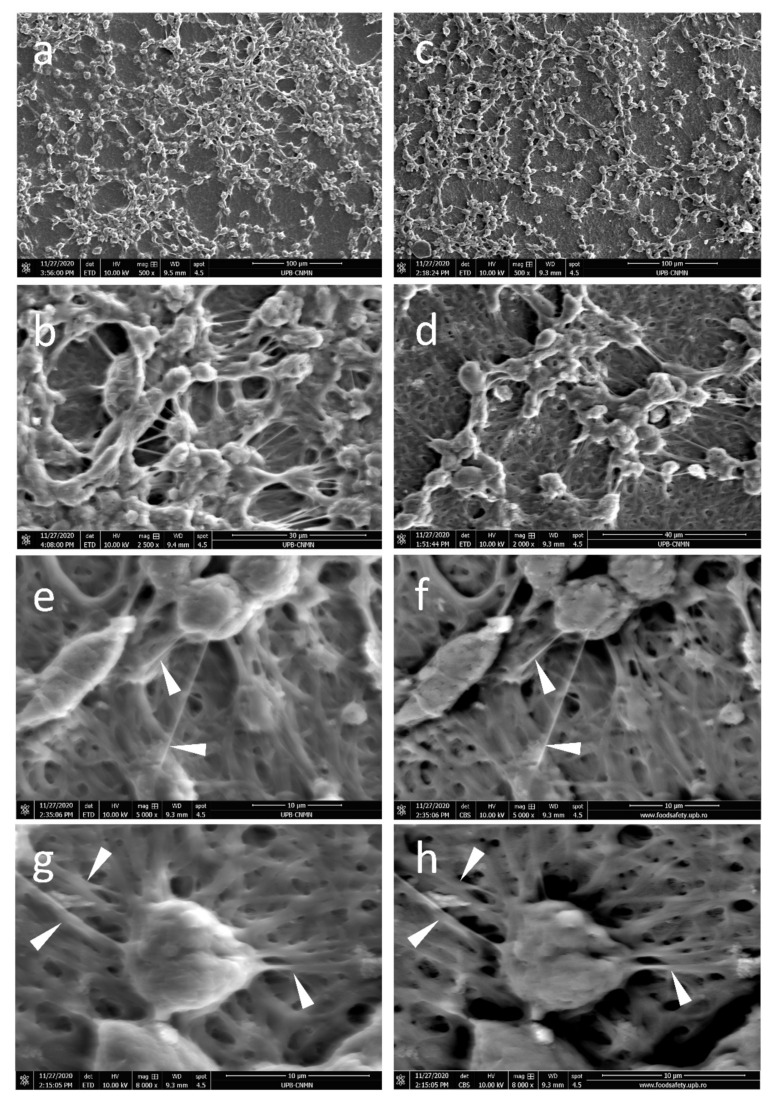
Representative SEM micrographs of NE-4C interactions with the fibrous scaffolds, at seven days post-seeding: Overview of NE-4C adhesion and spreading on (**a**,**b**) FG50, (**c**,**d**) FG50_NDs; NE-4C cells form well-defined cellular nanofibers (white arrowheads) to adhere onto the sub-micronic FG50_NDs fibers of the (**e**,**g**) ETD mode; (**f**,**h**) CBS mode.

Additional information on cell-scaffolds interactions was brought by the SEM micrographs. At seven days post culture, the FG50-based meshes are covered with numerous cells, as seen in Figure 5. Compact multicellular aggregates strongly adhere onto the FG50 substrate, with numerous cell–matrix and cell–cell interactions, while small areas remain uncovered by cells and few cells seem embedded into the surface layers of the FG50 scaffolds (Figure 5a). A multilayer cellular cluster with a strong nanofibers network developed for intercellular communication is visible in Figure 5b. While cells are randomly distributed on FG50, a certain regional distribution is noticed on FG50_NDs, with NE-4C forming longitudinal cords of about 100 µm long (Figure 5c,d). The robust cellular growth is accompanied by numerous intercellular interactions. Cellular nanofibers are firmly anchored onto the FG50_NDs substrate, forming a fiber-on-fiber construct (white arrows in Figure 5e–h).

**Figure 6:**
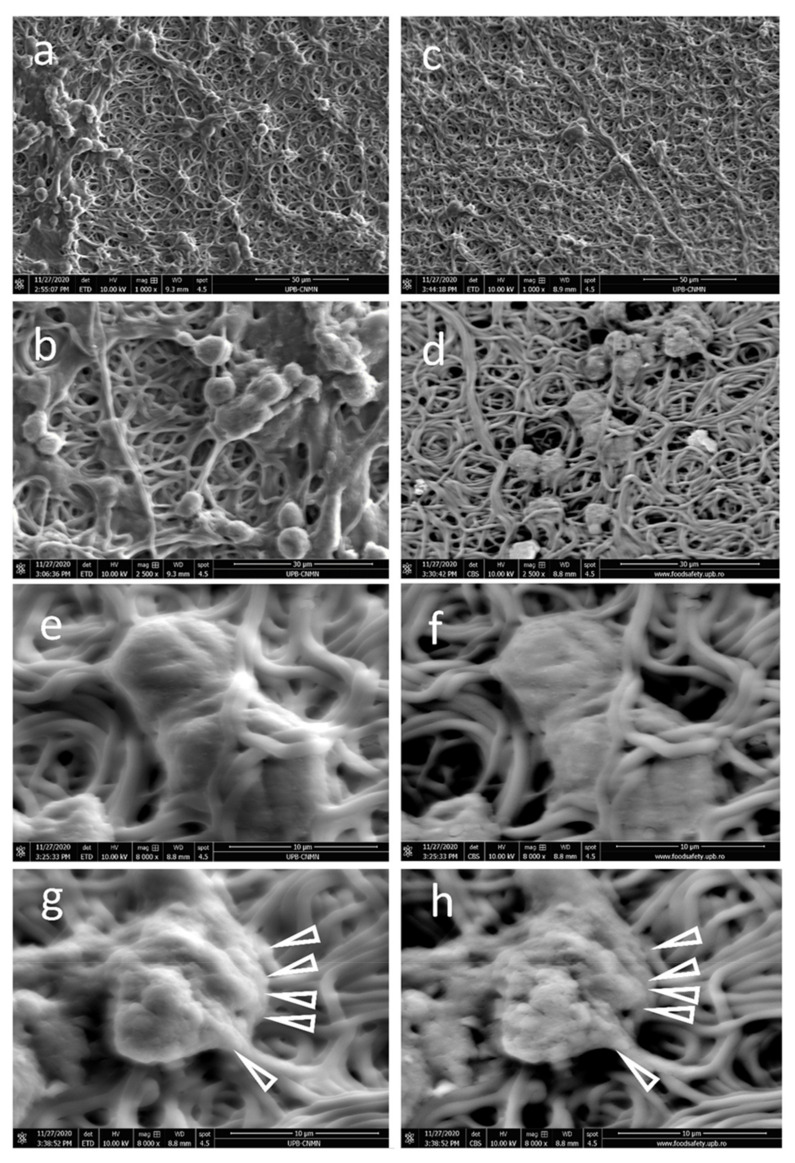
Representative SEM micrographs of NE-4C interactions with the fibrous scaffolds at seven days post-seeding: Overview of NE-4C adhesion on (**a**,**b**) FG70, (**c**,**d**) FG70_NDs; NE-4C cells colonize the fibrous FG70_NDs meshes infiltrating through the fibers to form (**e**,**g**) ETD mode; (**f**,**h**) CBS mode).

When enriching the fibers in gelatin, a different cellular distribution is noticed. NE-4C cells remain at the surface of FG70 fibers as compact cellular clusters with a certain regional orientation, probably favored by fiber bundles, as seen in Figure 6a,b. No individual nanofibers seem to be formed by cells adhering to the matrix when compared to the FG50 counterpart. Cells seem to start strongly embedding themselves into the fibrous scaffold. FG70_NDs scaffolds appear to stimulate intense cellular interactions. The cells are not visible at the surface, but rather infiltrating through the fibrous mesh, colonizing the deeper layers of the FG70_NDs scaffold (Figure 6c,d). Morphological details in Figure 6e,f are representative for cells-matrix interactions, which modified the geometry of the entangled polymer fibers, to generate a unique bio-construct with polymer fibers strongly attached to the cellular bodies. Other cell-scaffold specific features are evidenced in Figure 6e,f. Short and strong cellular adhesion pseudopodia are formed onto the FG70-NDs fibers. Such information is interesting, since it indicates the nanofiller-FG interactions specific to each series define the intensity of the cellular interphase.

## 4. Discussion

To date, the fabrication of nerve guidance conduits typically involved synthetic, nonbiodegradable tubular devices. Even though silicon tubes can lead to a successful repair, synthetic materials might eventually create other problems as a result of local fibrosis or compression [28]. Hence, researchers in the field focused on developments of natural biodegradable guidance channels, such as those based on gelatin [16,29]. FG is known to be a bio-friendly compound and used for various tissue engineering applications, among them bone tissue engineering [30], adipose tissue engineering [31], and nervous tissue engineering [32]. NDs, carbon-based nanomaterials, have lately emerged as a powerful tool for material functionalization [33]. The cytocompatibility of FG50 fibrous scaffold containing low amounts of NDs additive in contact with MG63 human osteosarcoma cell or human adipose-derived stem cells was previously reported as promising for a wide range of tissue engineering applications [23,24]. In this work, tubular scaffolds based on FG and low amounts of NDs were successfully fabricated using electrospinning. On the one hand, this study aimed to investigate the effect of NDs on the degradability and water affinity of the electrospun tubes. On the other hand, the interactions with NE-4C cells were tested to establish their potential in the field of peripheral nerve regeneration.

To prepare the electrospinning solutions, the 1% nanofiller dispersion is enriched with protein using two concentrations of FG, namely, 50% and 70%, respectively. In the electrospun fibers, FG macromolecules form a continuous hydrated matrix containing as second phase small NDs aggregates, potentially presenting interparticulate nanoscopically-confined FG molecular segments, also considering that NDs might act as nanoplatforms for adsorption of proteins. When NDs are introduced in the system, due to their high surface to volume ratio and functionalization with carboxyl groups, hydrogen bonding with gelatin molecules may lead to core-shell self-assembly, where the core is represented by NDs and the shell is generated by the protein macromolecules wrapping the nanoparticles. At the same time, significant interactions between ND-ND and protein-protein may occur through hydrogen bonding, as well as electrostatic forces. Thus, these core-shell aggregates managed by the interfacial interactions are unevenly distributed in electrospinning compositions. When fibers are created, a certain homogenous distribution is believed to be achieved through collector rotation and needle movement back and forward all along the collector length. When crosslinking takes place, the core-shell aggregates are covalently entrapped within fibers. The fact that fibers are crosslinked attached to the collector might have an influence over the flexibility of the polymeric structure as they cannot shrink as much as they would if they were crosslinked detached. Anyhow, the polymeric network became more rigid, due to the covalent bonds formed, and as it was revealed by dimensional stability evaluation, NDs decrease the length variation of the crosslinked scaffold when hydrated. NDs might act as an additional stabilizing nanoplatform improving the overall stability when the crosslinking is efficient (FG50_NDs).

We noticed that the higher the FG loading, the higher the diameter of the fibers, and for FG70_NDs, a tendency of local parallel orientation of the fiber segments forming regionally aligned bundles. This may suggest that both the distribution and orientation of the macromolecules and the physical interaction between NDs and FG depend on the nanofiller-to-protein ratio in the electrospinning solutions and further impact the properties of the nanocomposite. It was previously reported that modified mechanical properties in nanocomposites NDs—synthetic polymers were due to interactions between–COOH groups in NDs and OH- groups of the polymer, including suppression of segmental mobility [34,35]. Similarly, it may be speculated that the higher the FG content, the stronger the NDs-FG H-bonding leading to thicker fibers. Furthermore, higher swelling was recorded for FG70 and FG70_NDs when compared to FG50 series, the amount of protein significantly influencing the water affinity (Figure 1g, *p* < 0.001). It is unclear if this is due to ND-FG interactions or to a stronger FG crosslinking. These findings were completed by the interesting degradability results. It appears that overall, samples of FG70 series are less stable when compared to samples from FG50 series, despite the similar crosslinking treatment. Considering the larger diameters and the higher water retention of FG70 fibers, the diffusion of reactive GA molecules may be more difficult than in the thinner FG50 fibers leading to a looser network, providing a more biodegradable microenvironment. It is also possible that the NGs-FG phase is differently distributed and results in different degradation profiles. While interesting for nerve regeneration requiring mechanical support for approximately 100 days (as needed for a typical median or ulnar nerve injury of 100 mm, at an axonal regeneration rate in humans of 1 mm/day [36]), the FG_NDs tubular scaffolds would need further understanding and adjustment for the control of their stability.

Regarding the cellular interactions, in the present study, the meshes based on FG loaded with NDs were tested for their cytocompatibility. Our results highlight an impressively good interaction between neural progenitors and FG_NDs electrospun meshes. This is in agreement with other research that documented the biological effect of FG and NDs upon cellular behavior [37,38]. Thalhammer et al. have used NDs as a coating to enhance the formation of functional neuronal networks [39]. Their results indicated a good neuronal attachment, significant neurite outgrowth, and achievement of electrical networks. Soucy et al. have demonstrated that gelatin-based hydrogels supported in vitro Schwann cell proliferation and neurite outgrowth, becoming potential candidates for further clinical applications for peripheral nerve injuries [40].

One of the most interesting outcomes of the present study is that at 50% FG, cells remain at the surface of the scaffold, while FG70_NDs nanocomposites favor NE-4C infiltration into the fibrous mesh. Engineering fibrillar microenvironments are recognized as extremely important for steering cellular behavior [41,42]. It was reported that architectural remodeling by cells is favored by soft fibrous scaffolds, that allow fibers recruitment by cells leading to compacted fibers bundles [41]. In our study, SEM micrographs on FG70_NDs sample provide experimental evidence that cells remodeled the fibrillar scaffold migrating to the deeper layers. Only a few cells remain visible at the surface, partly covered by fibers (Figure 6d–h). This observation was also supported by confocal microscopy analysis, which identified numerous cells distributed within the scaffold, on many planes in FG70_NDs (Figure 4c). This interesting behavior will be further investigated. This is of major importance for the potential of such scaffolds in nerve repair and regeneration. Methods to enhance cell infiltration in fibrous materials are needed, as reported by Wu J. and Hong Y., who emphasized that most electrospun fibers are too dense and compact to provide cell colonization into the deeper layers [43]. An overview of approaches to increase the pores or to loosen the fibrous mesh is given in [43]. In our study, the most degradable fibers, FG70_NDs, behave as extracellular matrix-mimetic scaffolds allowing NE-4C cells to change scaffold geometry by moving the polymer fibers, while strong cell–fiber interactions occur.

## 5. Conclusions

To sum up, the FG_NDs loaded meshes developed in this study have been shown to support cell adhesion, growth, and proliferation. FG70_NDs presents the best cell-adhesion and provide cell infiltration, which is considered important for tissue repair and regeneration. They have not induced significant toxicity upon neural stem cells, which is an important feature for biomaterials, as they are most likely to not cause significant damage and immune response when implanted in vivo. While the four types of FG composites are validated for their cytocompatibility and can be further tested for in-depth tissue engineering approaches, FG70_NDs electrospun fibers seem to be the most ECM-mimetic and to open a new window in the development of biodegradable cell-interactive nanocomposites for nerve regeneration. Further investigation will be devoted to better understand the role of the NDs-polymer interface in modulating scaffolds properties, degradation, and cellular response.

## Data Availability

The data presented in this study are available on request from the corresponding author.
